# Reply to “Comment on Liu et al. ‘Discrepancies of Measured SAR between Traditional and Fast Measuring Systems’ *Int. J. Environ. Res. Public Health*, 2020, *17*, 2111”

**DOI:** 10.3390/ijerph17155355

**Published:** 2020-07-24

**Authors:** Zicheng Liu, Djamel Allal, Maurice Cox, Joe Wiart

**Affiliations:** 1 Chaire C2M, LTCI, Télécom Paris, 91120 Palaiseau, France; joe.wiart@telecom-paris.fr; 2 Laboratoire National de Métrologie et d’Essais, 78197 Trappes, France; djamel.allal@lne.fr; 3 National Physical Laboratory, Teddington TW11 0LW, UK; maurice.cox@npl.co.uk

**Keywords:** specific absorption rate, fast SAR measurement, field reconstruction, plane-wave expansion, traditional SAR measurement, measurement discrepancy

## Abstract

The authors of this reply published an article in *International Journal of Environmental Research and Public Health* and received comments from Douglas and Kuster. Responses are made to these comments with complementary explanations and numerical results.

## 1. Introduction

The authors of this reply published an article [1] in *International Journal of Environmental Research and Public Health* that studies discrepancies of estimated SAR by traditional and fast SAR measuring systems. The traditional system applies two steps, area scan and zoom scan, that can be time consuming. Moreover, fast measuring systems fabricated by different manufacturers reportedly yield inconsistent estimations of SAR [2]. The authors made efforts to analyze these phenomena by simulating the measurement, where the concerned fast measuring system reconstructs the near field based on measured electric fields (amplitude and phase) on a plane inside the phantom.

Detailed knowledge of key components such as the reconstruction algorithms adopted by the implemented systems was unavailable. Hence, the analysis presented was based on commonly used settings in the literature. The authors consequently tried to give some insight (rather than a conclusion) on estimation discrepancies.

The authors received valuable comments [3] from Douglas and Kuster about this article. The comments gave opinions about the concerned fast measuring system citing the new standard IEC 62209-2:AMD1 [4] concerned with the traditional measuring system. The comments also revealed that the probes used by Douglas and Kuster can be at a distance of 1.4 mm to the phantom surface. Here, a response from the authors of [1] is given.

## 2. Reply to Comments

The comments are divided into two parts, which are for the presented results of the concerned fast measuring system on the one hand and the traditional system on the other. Accordingly, the reply is given in two subsections.

### 2.1. Fast Measuring System

The comments mentioned that as a potentially large uncertainty source, field distortion due to the array is not addressed in our article. The authors agree that factors including the dielectric enclosure of probes lead to distorted fields and the associated uncertainty is essential to quantify fully the estimation accuracy. The related uncertainty analysis is absent due to the lack of mathematical modelling tools (such as for determining the distribution of measurement errors due to field distortion). However, even without the uncertainty from field distortion, the analysis of other factors including probe position, permittivity and conductivity of the phantom, measurement accuracy, and coupling effects already reveals the ill-conditioned nature of the problem of field reconstruction and the resulting trade-off between estimation precision and estimation reliability.

The comments mentioned that the filtering coefficient (denoted as δ) cannot be known a priori and must be chosen arbitrarily. A larger δ means a greater number of evanescent components are reconstructed and the system has the potential to yield more accurate estimations. However, the decaying propagation of evanescent waves leads to an ill-conditioned problem in field reconstructions. Small uncertainties in the measured fields may cause a large uncertainty in estimated SAR. Therefore, the selection of δ is related to the trade off between accuracy and reliability. As mentioned in the article, developing advanced reconstruction algorithms is not covered for the reason given in Section 1. Thus, the authors did not attempt to propose a process to select the optimal δ. However, the authors believe that the chosen value is strongly related to the wave frequency and the electromagnetic properties of the phantom. When the frequency is higher or the phantom medium is more lossy, the decaying rate of evanescent components is faster and a smaller value of δ is preferred to avoid large estimation uncertainty. Consequently, it is possible to optimize the value of δ according to the configuration of tested devices and measured fields.

The comments mentioned the errors due to non-zero SAR at the array edges were not examined in the paper. As illustrated in the paper, the method of plane-wave expansion assumes that the amplitude of electric fields outside the measurement domain is quite small. The violation of this assumption would yield greater reconstruction errors and the estimation of SAR would be biased. The well estimated values of SAR for the 11 cases in Figure 3 and Figure 4 (in the article [1]) indicate that the non-zero SAR at the array edges does not greatly influence the performance of the reconstruction algorithm.

Here, the authors emphasize that the analysis of the fast measuring system based on field reconstructions by the method of plane-wave expansion is to give insights on discrepancies between fast measuring systems produced by different manufacturers. However, since the applied reconstruction algorithm (which may be sophisticated or follow different reconstruction methodologies) in the actual systems is inaccessible, the presented results may be inconsistent with the performance of actual systems.

### 2.2. Traditional Measuring System

The comments mentioned linear interpolation and extrapolation are applied in the paper despite the standard clearly recommending more accurate interpolation and extrapolation methods that use splines and polynomials. Different measuring systems for estimating SAR in the traditional way may use different interpolation and extrapolation algorithms, which would influence the estimation performance. To show the effects, since the applied algorithms in actual systems are inaccessible, the results of linear interpolation and extrapolation are presented to make comparisons with results of splines. However, that does not mean (and the authors did not claim) that linear algorithms are recommended or used in actual systems. The presented results actually showed that more accurate estimations were obtained with splines.

The comments mentioned the new standard IEC 62209-2 AMD1 has stricter scanning requirements for sources with strong field decay and state-of-the-art probe scanning measurement systems enable measurements as close as 1.4 mm from the surface. The authors agree that in the new standard, stricter scanning requirements are given. For frequencies above 3 GHz, the grid step in the vertical direction shall be ≤10/(f[GHz]−1) mm [4] clause 6.3.1 for uniform grids. Complementary results for the traditional measuring system are given with the new requirement.

**Figure 1:**
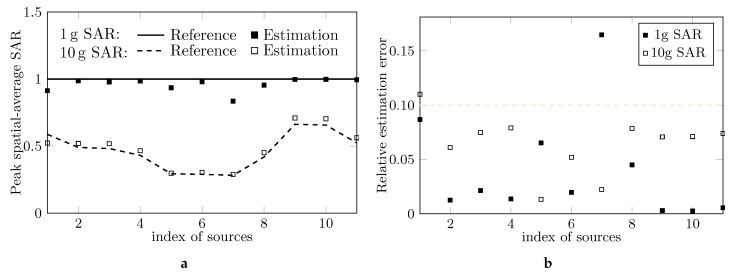
(**a**) Peak spatial-average SAR estimated by the traditional SAR measuring system with the setting in Table 1 and (**b**) associated relative estimation error.

With the frequency-dependent setting of the simulations in Table 1, the estimations of peak spatial-average SAR are given in Figure 1a and the associated relative estimation errors in Figure 1b. Splines are used for interpolation and extrapolation. The magnitudes of the relative errors are below 10% for all cases except the estimated 10 g SAR for the 1st case and 1 g SAR for the 7th case, the frequencies of which being 850 MHz and 750 MHz, respectively.

**Figure 2:**
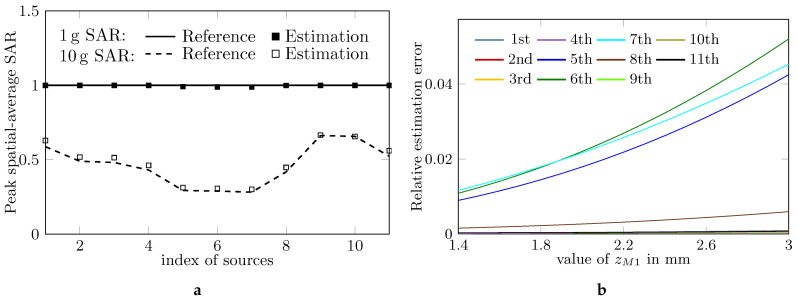
Setting horizontal and vertical grid spacing as 4 mm and 2 mm, respectively, (**a**) shows estimated 1 g and 10 g SAR when $z_{M1}$ = 1.4 mm, (**b**) the relative estimation error with increasing $z_{M1}$.

The asserted distance 1.4 mm is actually much smaller than the required $z_{M1}$ in [4], the maximum distance between probes and the phantom surface. Here, the effects of the maximum distance between probes and the phantom surface are studied by setting the same $z_{M1}$ for the 11 cases and increasing the value of $z_{M1}$ from 1.4 mm to 3 mm with the step 0.1 mm. The other settings in Table 1 are applied, except that the horizontal grid spacing in zoom scan is chosen as 4 mm, which was claimed as the setting of DASY6 [3] and the vertical grid spacing equals 2 mm to satisfy the requirement of IEC 62209-2 AMD1 for the 11 cases. When $z_{M1}=$ 1.4 mm, the estimated values of peak spatial-average SAR are given in Figure 2a. All relative estimation errors are below 10% in magnitude. Figure 2b shows that the relative estimation errors for 1g SAR increase with $z_{M1}$ and the rate of increase is high for the 5th, 6th and 7th cases. The observations indicate that the estimation accuracy of the traditional SAR measuring system is very sensitive to the value of $z_{M1}$.

## Figures and Tables

**Table 1:** Settings for estimations in Figure 1.

**Area Scan**	scan size	100 mm × 100 mm × 30 mm
	horizontal grid spacing	uniform grids with step 10 mm
	vertical grid spacing	uniform grids with step 10 mm
	maximum distance between probe and surface of phantom	2.1 mm for 5th case, 1.9 mm for 6th case, 5.0 mm for the other cases
**Zoom Scan**	scan size	30 mm × 30 mm × 30 mm
	horizontal grid spacing	uniform grids with step 4.3 mm for 5th case, 4.1 mm for 6th case, 8.0 mm for the other cases
	vertical grid spacing	uniform grids with step 2.2 mm for 5th case, 2.0 mm for 6th case, 5.0 mm for the other cases
	maximum distance between probe and surface of phantom	2.1 mm for 5th case, 1.9 mm for 6th case, 5.0 mm for the other cases
**Interpolation & Extrapolation**	horizontal grid spacing	1 mm
	vertical grid spacing	1 mm
